# Interaction of Physical Activity, Sleep, and Cognitive Function in Stroke: SEM and ML Approaches

**DOI:** 10.1093/geroni/igaf122.1916

**Published:** 2025-12-31

**Authors:** Mo Yi, Lan Gao, Li Chen, Junxin Li, Tangsheng Zhong

**Affiliations:** Peking University, Beijing, Beijing, China (People’s Republic); First Hospital of Jilin University, Changchun, Jilin, China (People’s Republic); Jilin University, Changchun, Jilin, China (People’s Republic); Johns Hopkins University, Baltimore, Maryland, United States; First Hospital of Jilin University, Changchun, Jilin, China (People’s Republic)

## Abstract

Cognitive impairment is prevalent among stroke survivors, highlighting the importance of exploring modifiable lifestyle factors such as physical activity and sleep, along with their biological underpinnings. This study aimed to investigate how physical activity and sleep influence cognitive function in stroke survivors, examining potential biological mediators. Cross-sectional data collected at hospital admission from 262 stroke survivors (First Hospital of Jilin University, 2024) were analyzed. Measures included self-reported weekly physical activity (exercise frequency/duration), nighttime sleep (duration, latency), cognitive function (Montreal Cognitive Assessment [MOCA]), emotional status (Hamilton Depression Rating Scale [HAMD]), and Serum neurological (homocysteine [Hcy]) and inflammatory (hs-CRP) biomarkers. Participants were aged 65.4±8.2 years, 43% female, and 58% showed cognitive impairment. Correlation and regression analyses examined associations, and structural equation modeling (SEM) explored causal pathways. Machine learning (ML) methods identified key predictors of cognitive impairment. Frequent physical activity (r = 0.35, p < 0.001) and adequate sleep (r = 0.25, p < 0.001) significantly correlated with higher cognitive scores. Depression severity (r=-0.40, p < 0.001) and elevated Hcy (r=-0.36, p < 0.001) negatively correlated with cognition. Regression analyses confirmed exercise frequency (β = 0.34, p < 0.001) and sleep duration (β = 0.29, p < 0.001) as independent positive predictors of cognitive function. SEM demonstrated direct beneficial effects of physical activity (β = 0.41, p < 0.001) on cognition and indirect effects mediated by improved sleep and reduced inflammation (CFI>0.9). ML predicted cognitive impairment (accuracy>80%), identifying age, education, Hcy level, physical activity, and sleep as crucial predictors. Physical activity and sleep synergistically enhance cognition in stroke survivors via inflammatory and neurological pathways. Promoting regular exercise, optimizing sleep, and managing biomarkers may preserve cognitive function.

